# Multi-Task Neural Networks and Molecular Fingerprints to Enhance Compound Identification from LC-MS/MS Data

**DOI:** 10.3390/molecules27185827

**Published:** 2022-09-08

**Authors:** Viviana Consonni, Fabio Gosetti, Veronica Termopoli, Roberto Todeschini, Cecile Valsecchi, Davide Ballabio

**Affiliations:** Department of Earth and Environmental Sciences, University of Milano-Bicocca, Piazza della Scienza 1, 20126 Milano, Italy

**Keywords:** LC-MS/MS, chemometrics, fingerprints, similarity matching, classification, neural networks, multi-task

## Abstract

Mass spectrometry (MS) is widely used for the identification of chemical compounds by matching the experimentally acquired mass spectrum against a database of reference spectra. However, this approach suffers from a limited coverage of the existing databases causing a failure in the identification of a compound not present in the database. Among the computational approaches for mining metabolite structures based on MS data, one option is to predict molecular fingerprints from the mass spectra by means of chemometric strategies and then use them to screen compound libraries. This can be carried out by calibrating multi-task artificial neural networks from large datasets of mass spectra, used as inputs, and molecular fingerprints as outputs. In this study, we prepared a large LC-MS/MS dataset from an on-line open repository. These data were used to train and evaluate deep-learning-based approaches to predict molecular fingerprints and retrieve the structure of unknown compounds from their LC-MS/MS spectra. Effects of data sparseness and the impact of different strategies of data curing and dimensionality reduction on the output accuracy have been evaluated. Moreover, extensive diagnostics have been carried out to evaluate modelling advantages and drawbacks as a function of the explored chemical space.

## 1. Introduction

Mass spectrometry (MS) is a commonly used detection analytical technique for the identification of compounds in food, environmental, biological and forensic samples [1,2,3]. It is generally coupled with liquid chromatography (LC) or gas chromatography (GC), depending on the type of sample to be analysed. GC-MS uses the most popular hard-ionisation technique (Electron Ionisation, EI), which generates a unique MS spectrum characterised by extensive fragmentation: this is specific for the target compound and useful for its structure elucidation [4]. Since the EI source works in high-vacuum conditions, the EI-based MS spectrum is independent of the gas chromatographic conditions and represents a chemical identifier of the investigated molecule with high accuracy. Conversely, in LC-MS the ionisation process occurs at atmospheric pressure conditions, and this leads to MS spectra that cannot unequivocally characterise the compound, since the acquisition of these spectra depends on several experimental factors, such as source type and mechanism of ionisation, organic solvent, potential matrix effects, presence of additives and their concentration in the mobile phase [5]. In tandem mass spectrometry (MS/MS), a precursor ion is fragmented in a collision cell to generate a product ion spectrum (MS/MS spectrum) to get measurements which are more independent of the chromatographic conditions [6]. Sometimes, these second-level ions can be fragmented even further, giving MS3 spectra and so on. Ad hoc libraries for the recognition of MS/MS spectra can be found on the market. However, the obtained MS/MS spectra are still dependent on the collision gas, collision process, and collision energy involved in the fragmentation process. Therefore, a plethora of different MS spectra can be related to the same compound, from little or no fragmentation, in which the precursor ion is still present, to highly fragmented spectra [7].

Small molecules (below 1500 Da), which form as intermediates and products of all chemical reactions within cells of living organisms, are called metabolites. Metabolites cover a wide range of compound classes and their structural diversity is very large, despite their small size. In metabolomics, the high-throughput characterisation of metabolites present in a biological sample is increasingly important across biomedical and life sciences [8]. A commonly employed analytical platform for metabolomic studies includes LC-MS, due to its ability to analyse a sizable number of metabolites with a limited amount of biological material compared to other platforms.

Identification of chemical compounds through MS/MS spectra is thus a prerequisite for further data interpretation and it is probably the most time-consuming and laborious step in metabolomics experiments [9,10,11,12]. Metabolite identification requires matching the observed spectrum against a database of reference spectra originating from similar equipment and closely matching operating parameters, a condition that is rarely satisfied in public repositories. Furthermore, the computational support for identification of metabolites not present in reference databases is lacking [13]. The most common routine method implies spectral matching. In particular, it calculates the similarities between the spectrum of an unknown compound and the spectra of standards in the database and the structure of the standard with the highest similarity is predicted as the structure of the unknown. Though widely used, this approach suffers from a coverage problem: if an unknown compound is not in the database, it can never be identified [4]. Despite the intense ongoing efforts to map the metabolome of various organisms, existing databases cover only a small percentage of the actual metabolites that occur in organisms. It is estimated that less than 10% of metabolites have experimental reference mass spectra [14], while only a small fraction of known compounds has its curated reference MS/MS spectra in these spectral libraries: it has been estimated that “only 1.8% of spectra in an untargeted metabolomics experiment can be annotated” [15]. Thus, the ability to annotate ‘known unknowns’ through MS/MS spectral matching is largely limited [16]. Such a limitation requires developing novel methods to fill in the gap between existing experimental spectra and spectra absent from libraries [12].

To overcome this limitation of the spectral matching, different computational approaches for mining metabolite structures based on MS data have been developed [17]. The rule-based fragmentation spectrum prediction applies fragmentation rules to a set of candidate molecular structures, which are then matched to the measured fragmentation spectrum. Approaches based on combinatorial fragmentation compute all possible fragments of a candidate structure by systematically breaking bonds to explain the peaks in the measured spectrum [18,19]. Competitive fragmentation modelling is another approach [20] that predicts peaks that are most likely to be observed by means of a probabilistic generative model for the fragmentation process, which learns from experimental data. 

Alternative computational methods for metabolite annotation do not directly learn a relationship between the spectrum and the metabolite; instead, they predict molecular fingerprints from MS spectra by means of machine learning strategies, which are later used to identify metabolites [12]. In fact, the recent availability of large public mass spectral databases enabled the development of empirical mathematical models to predict structural properties from mass spectra [13]. Basically, the presence or absence of structural features or substructures of each chemical compound can be represented by a set of binary bits, which are organised in a vector defining the molecular fingerprint [21,22]. The fingerprint of an unknown compound can be predicted from its MS spectrum using a trained machine learning method, which must be calibrated on large datasets of mass spectra (used as inputs) and molecular fingerprints (as outputs) [4,13]. Therefore, the predicted fingerprint can be matched against a very large database of chemical structures, thus enhancing the recognition of metabolites, which may not even be present in the reference MS spectral database. Similarity scores between the predicted fingerprint and those of the candidate compounds can be ranked so that the candidates with the highest rank can be used for annotation [12].

In this study, we propose a chemometric approach to predict molecular fingerprints and retrieve the structure of unknown compounds from their LC-MS/MS spectra. We have evaluated and compared different strategies to enhance the output accuracy by taking into account different curing solutions, sparseness conditions and methods for data dimensionality reduction. Moreover, solutions to tune the neural network hyperparameters by avoiding potential overfitting and improving the similarity matching between predicted and experimental fingerprints have been adopted. Finally, we explored the modelling outputs with extensive diagnostic tools to evaluate advantages and drawbacks as a function of the explored chemical space.

## 2. Materials and Methods

### 2.1. Data Collection, Curing and Dimensionality Reduction

We collected 138,225 LC-MS/MS spectra from the MassBank of North America (MoNA) database [23]. MoNA is a repository of mass spectra records derived from collaborative efforts, making it one of the largest archives of freely available spectra, although this involves careful pre-processing of spectra to standardise them and exclude anomalous spectra. 

Each spectrum (i.e., pairs of peaks and abundances) is accompanied by metadata with information on the compound (e.g., chemical name and molecular structure) and the experimental conditions used for the spectrum measurement (i.e., the type of instrument, the precursor and collision energy). When needed, the terminology of the experimental conditions was standardised: for example, the instrument type field for different spectra contained both “ESI-QFT” and “LC-ESI-QFT” values, which are referred to the same instrument and thus uniformed to “LC-ESI-QFT”.

To get input data with reasonable quality for the subsequent modelling task, we cured the collected database and initially applied a pre-processing workflow solely based on the metadata. In particular, we applied the following criteria to discard:

1. records with missing instrument and/or collision energy annotations;
2. ambiguous records with collision energy units that were not compatible with the matched instrument type;
3. records with spectra acquired in negative ionisation mode;
4. records showing inconsistency between MW and precursor ions;
5. records with spectra acquired with ion trap instrumentation;
6. records with spectra acquired with collision energy values (CE) lower than 5 V or higher than 70 V;
7. records with spectra measured with Atmospheric Pressure Photo-Ionisation (APPI), Atmospheric Pressure Chemical-Ionisation (APCI), Linear Trap (LT) and Orbitrap, due to the few entries available in the database;
8. records with precursor ions different from the most common ones, such as [M+H]^+^, [M+Na]^+^, [M+K]^+^, and [M+NH4]^+^.

We further processed the database on the basis of MS spectral information by:

1. discarding records with spectra showing less than two peaks;
2. for each spectrum, removing peaks at *m*/*z* higher than the precursor ions.

After these curing actions, we obtained a first dataset with 40,571 spectra for 5557 different molecules: more spectra (measured in different experimental conditions) could be associated with the same chemical compound. In order to evaluate the effect of the quality of data to be used as input to train the chemometric approaches, we further cured the MS data by discarding 1) spectra with precursor ions different from [M+H]^+^ and 2) spectra with less than five peaks, in order to increase the amount of peak information for the model training. By applying these additional curing actions, we obtained a second reduced dataset with 12,550 spectra.

Afterwards, the length of the spectral vectors was standardised to organise the MS spectra in a suitable dataset to be used as input for the modelling phase. The dataset was structured as a data matrix **X**, where each row represents a spectrum and each column an *m*/*z* value; thus, each entry *x_ij_* of the matrix is the signal measured at the *j*-th *m*/*z* for the *i*-th spectrum. The considered *m*/*z* range was defined between 45.0 Da and 704.5 Da, being the most comprehensive *m*/*z* range considering all the available spectra. The resolution was defined at one decimal point to reduce data sparseness and get a reasonable data size, leading, therefore, to spectral vectors of size 6596 bits. Since the resolution of the original data was higher than 1 decimal point, the intensity of each bit was set to the maximum among all fragments that had the same mass when considering only one decimal point. This preprocessing ensured that in each spectrum we had at least one peak with intensity equal to 100. Finally, for each spectrum, the intensity values were standardised by dividing by the maximum intensity (100) to obtain an appropriate scale for the subsequent modelling through artificial neural networks.

The final cured MS data were thus organised in two datasets (i.e., data matrices) with dimensions 40,571 rows (40K dataset) and 12,550 rows (12K dataset) times 6596 columns. The MS data are available for download at the Milano Chemometrics and QSAR Research group [24].

The MS spectra in analysis have a median number of peaks equal to 81, meaning that the majority of the 6596 bits of the spectral vectors are equal to zero. As a consequence, both the 12K and 40K datasets are very sparse: 99.3% and 97.1% of the elements of the data matrices are equal to zero, respectively. In order to enhance the subsequent learning by means of artificial neural networks (ANN), we reduced data sparseness by applying a data reduction method. In this way, we retained the majority of data variance in a lower dimensional space and at the same time reduced the amount of sparseness in the dataset. 

Specifically, we calculated Sparse Principal Component Analysis (SPCA) and used the scores as input variables for the multi-task modelling based on neural networks. Principal Component Analysis (PCA) is a linear transformation that maximises the variance of the projected data and produces a more compact data representation. SPCA aims at maintaining the properties of PCA, such as maximisation of variance and uncorrelated components, but enforces sparsity of the loadings. Therefore, each principal component is a linear combination of few of the original variables and this reflects the original MS data structure [25,26].

The number of significant components to be selected was defined on the basis of the cumulative explained variance. In particular, for the 40K dataset, we found two solutions to approximate the original data by retaining 500 and 1000 components, which explained 84.3% and 94.2% of the total data variance, respectively. For the 12K dataset, 500 components were retained, explaining 93.1% of the variance. These amounts of information were considered sufficiently high to adequately represent the original data.

### 2.2. Molecular Fingerprints (FPs)

The molecular structures of the compounds were encoded by the MACCS keys [27], which are molecular fingerprints suitable for the ANNs training. MACCS keys are binary vectors of predefined length, where each of the 166 bits is associated with a substructural pattern. MACCS keys characterise the molecular structures in a simple way and narrow down the feature/task space, reducing the data sparseness. Moreover, the same fingerprint representation has already been used alone or in combination with other descriptors in similar works [4,12,14].

**Figure 1:**
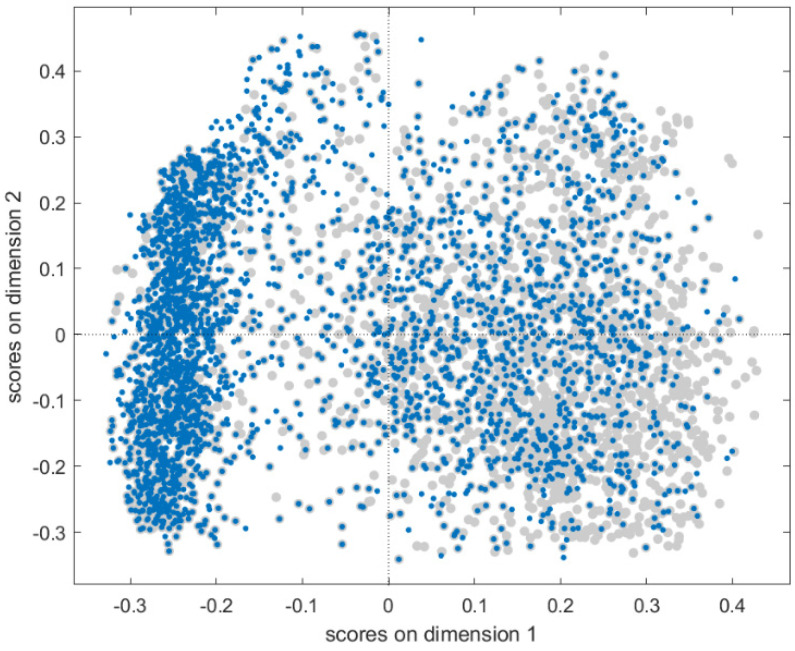
Score plot of the first and second MDS coordinates for the molecular fingerprints. Fingerprints of the 12K and 40K datasets are coloured in blue and grey, respectively.

Fingerprints were added to the spectral datasets as compound identifiers, resulting in a total of 166 additional columns to be used as output (responses) for the multi-task modelling phase. The chemical spaces of the two datasets have been represented through Multidimensional Scaling (MDS, Jaccard-Tanimoto similarity) to evaluate the degree of overlapping of the smallest cured set (12K) with respect to the 40K dataset. The scores of the first two MDS coordinates are plotted in Figure 1, where both datasets span over similar structural spaces, as represented by fingerprints. The fingerprint dataset is available for download at the Milano Chemometrics and QSAR Research group [24].

### 2.3. Multi-Task Modelling

Multi-task ANNs were used to learn and predict molecular fingerprints from LC-MS/MS spectra. In fact, the fingerprints include 166 binary bits, each bit corresponding to a specific task. Instead of training 166 bit-specific classifiers, we exploited the multi-task learning paradigm, which allows the simultaneous prediction of joint tasks. Usually, multi-task ANNs consist of fully connected layers with as many neurons in the output layer as the number of tasks [28,29,30]. When some dependence relationships exist among the tasks, the model should learn a joint representation of these tasks. Since the fingerprint bits encode existing chemical features, relationships among bits can be assumed.

#### 2.3.1. Artificial Neural Networks (ANNs)

ANNs are nonlinear modelling strategies that perform repeated linear and nonlinear transformations on their input [31,32]. The basic unit of ANNs are neurons or nodes arranged in layers. In the simplest case, each neuron is connected to all the neurons of the subsequent layer. Each neuron corresponds to an activation function, while the connections represent weights.

ANNs are trained for a number of iterations (epochs), which usually consist of a feedforward phase followed by a backpropagation phase. In the feedforward phase, the output of each layer is computed. Usually, at the beginning of the training, weights are randomly initialised. Then, for each input, the predicted output is compared to the observed value by means of a loss function. Through the computation of the derivatives of the loss and activation functions (i.e., gradients), all the weights are updated in a backpropagation manner, to minimise the loss function. The updating step width can be modulated by a tunable value called learning rate. For computational and learning efficiency, in each training step (i.e., iteration) a subset of training objects, called batch, is used. When all the objects are seen by the model (i.e., after a number of iterations equal to the training set size divided by the batch size), a training epoch is completed. Furthermore, several strategies (i.e., regularisation techniques) can be used to improve the network’s generalising ability and reduce overfitting. This is the case of dropout, weight decay (Ridge L1 or Lasso L2 regularisation) and early stopping [33,34,35,36]. In particular, training can be automatically terminated when no improvement in performance metrics is observed for a predefined number of epochs (called patience) [36].

A multi-task ANN includes as many nodes in the output layer as the number of tasks to be predicted [37]. It is also possible to add other single-task specific hidden layers before the task-specific classifier. In other words, the input to hidden, and eventually some hidden to hidden, weights are shared by all tasks, whereas the hidden to output, and eventually some hidden-to-hidden weights, are task-dependent [38].

We used the sigmoid as the activation function for the nodes in the output layer since each node must be linked to the prediction of a binary value, i.e., our tasks are the binary bits of the fingerprints. The output of the sigmoid function is a probability and consequently predictions of the multi-task network will not have the form (0,0,…,1,…0), but values in the range [0, 1]. Therefore, we applied a threshold to each output node (i.e., bit) to define the binary predicted value. The intuition is that different bits are represented differently by the training data and, therefore, the network may not be equally sensitive to all of them. Thresholds were estimated on the basis of the Bayes theorem by minimising the number of false positives (0 bits predicted as 1) and false negatives (1 bits predicted as 0) [39].

To reduce the effect of the initial random weight initialisation on the prediction performance, we trained each network five times and then we computed the final bit prediction on the basis of a majority voting approach. Thus, each single bit was predicted as 0 or 1 by combining the predictions provided by the five independent replicates and labelling the bit with the most frequently predicted value among 0 and 1 [40].

#### 2.3.2. Validation Protocol

Spectra were randomly divided into training (72%), validation (15%) and test (13%) sets. As previously described, in our dataset different MS spectra can be associated with the same chemical compound and thus with the same fingerprint. Therefore, in order to stress the validation conditions and use a strict protocol to avoid optimistic results in terms of prediction accuracy, we avoided the selection of the spectra associated with the same molecule in different sets.

In other words, all of the MS spectra of the same compound were included only in one of the three sets (training, validation or test). In this way, the prediction of unknown compounds can be better simulated in the validation phase. Training spectra were used for the ANN learning phase, validation spectra for the optimisation of the network’s architecture, while spectra in the test set were never used for training or tuning but just for the final validation of the model predictive ability. Table 1 reports the number of spectra and compounds included in the training, validation and test sets for both the 40K and 12K datasets.

#### 2.3.3. Performance Measures for Multi-Task Modelling and Similarity Matching

The prediction accuracy of ANNs was assessed through both similarity matching indices for binary fingerprints and classical classification measures, which were adapted for the multi-task modelling [41,42]. In particular, the average Non-Error Rate (*NER*) was used to estimate the overall capability of the models to correctly predict the binary output of all the tasks:

(1) $$NER=\overset{T}{\underset{t=1}{\sum }}NER_{t}$$

where *T* is the number of tasks (166 in our case) and *NER_t_* is the Non-Error Rate achieved on the *t*-th task, which is defined as:

(2) $$NER_{t}=\frac{Sn_{t}+Sp_{t}}{2}$$

where *Sn_t_* and *Sp_t_* represent the sensitivity and specificity for the *t*-th task, respectively, and are calculated as follows:

(3) $$Sn_{t}=\frac{TP_{t}}{Tp_{t}+FN_{t}}$$

(4) $$Sp_{t}=\frac{TN_{t}}{TN_{t}+FP_{t}}$$

*TP_t_*, *TN_t_, FP_t_* and *FN_t_* are the number of true positive (bits = 1 correctly predicted), true negative (bits = 0 correctly predicted), false positive (bits = 0 erroneously predicted as 1) and false negative (bits = 1 erroneously predicted as 0) for the *t*-th task. Therefore, the higher the sensitivity and specificity, the higher *NER* and the better the model in terms of prediction accuracy. On the other hand, the percentage of correctly predicted bits was not used as a measure to assess the model quality, because this index is known to be biased when dealing with unbalanced classification tasks [41]; this was the case, the molecular fingerprints being very sparse and, thus, characterised by a very high percentage of zero values (77%).

Rather than the accurate prediction of the single bits of molecular fingerprints, the main objective of our modelling approach was to maximise the similarity between predicted and experimental fingerprints to enhance the identification of unknown metabolites when matching the predicted fingerprints against molecular databases that include candidate compounds for annotation.

We used the Jaccard–Tanimoto similarity index (*JT_i_*) to evaluate the similarity between the true fingerprint of the compound and the fingerprint predicted by the multi-task model from the corresponding MS spectrum [43,44], as follows:

(5) $$JT_{i}=\frac{a_{i}}{a_{i}+b_{i}+c_{i}}$$

where, for the *i*-th spectrum, *a_i_* is a measure of similarity and corresponds to the number of common bits equal to 1 for both the true and predicted fingerprints, *b_i_* is a measure of dissimilarity and is equal to the number of bits equal to 1 for the true and 0 for the predicted fingerprint, *c_i_* is again a measure of dissimilarity and is equal to the number of bits equal to 0 for the true and 1 for the predicted fingerprint, respectively. Then, average similarities can be calculated over all the *JT_i_* values of specific sets of spectra.

#### 2.3.4. Tuning of Artificial Neural Network Hyperparameters

We fine-tuned the ANNs hyperparameters by means of a Tree-structured Parzen Estimator (TPE) [45,46], which approximates the performance of hyperparameters on the basis of historical measurements and then subsequently chooses new values of hyperparameters to test. The TPE approach models P(x|y) and P(y) following the Bayesian rule, where x represents the hyperparameters and y the associated quality score. In other words, the TPE approach formulates the hyperparameter optimisation as a process of maximising (or minimising) an objective function *F* that takes a set of hyperparameters as an input and returns its quality score. As optimisation criterion, we used the following *F* objective function (to be maximised):

(6) $$F=JT_{val}\cdot \left(1-\left|JT_{train}-JT_{val}\right|\right)$$

where *JT_val_* and *JT_train_* are the average Jaccard–Tanimoto similarity indices for the validation and training sets, respectively. In this way, we maximised the similarity between the predicted and true fingerprints for the validation set, but, at the same time, we minimised the potential overfitting, trying to minimise the difference between performance on the training and validation sets.

### 2.4. Software and Code

The calculation of MACCS keys fingerprints was carried out by means of the “rdkit.Chem.MACCSkeys” module and the “GenMACCSKeys” function [47]. The multi-task ANNs were calculated in Python 3.6.4 [48] using the Keras 2.4.3 [49] package with TensorFlow 2.4.1 [50] backend. The tuning of hyperparameters was carried out by means of the Optuna 2.10.0 package in Python [45]. Diagnostic of the ANNs outputs was carried out by means of the PCA toolbox for MATLAB [51]. Sparse PCA was calculated through the SpasSM Matlab toolbox [26]. Violin plots were created with the code available at [52].

## 3. Results

### 3.1. Optimisation of the Multi-Task Model

We tested different network architectures through the TPE approach. We initially defined the hyperparameters space by setting the minimum and maximum levels for each quantitative (i.e., number of neurons, number of task-specific neurons, learning rate, dropout, batch size, patience) and qualitative hyperparameter (i.e., activation function and optimisation type), which are collected in Table 2.

Then, we let the TPE approach explore the solution space with 100 iterations and select as optimal those solutions associated with the highest objective function, that is, architectures which could maximise the similarity between predicted and true fingerprints for the validation set and contemporaneously minimise the potential overfitting.

For both datasets (40K and 12K), the best architecture was composed by one layer of 100 neurons, with sigmoid activation function, dropout around 0.30, RMSprop as optimisation function, intermediate values for learning rate (0.006 and 0.0025, respectively), similar number of epochs (expressed as patience, 95 and 114) and a different number of task-specific neurons (250 and 500), as shown in Table 2. Thus, regardless of the data size, ANNs had similar architectures to better model the data.

**Figure 2:**
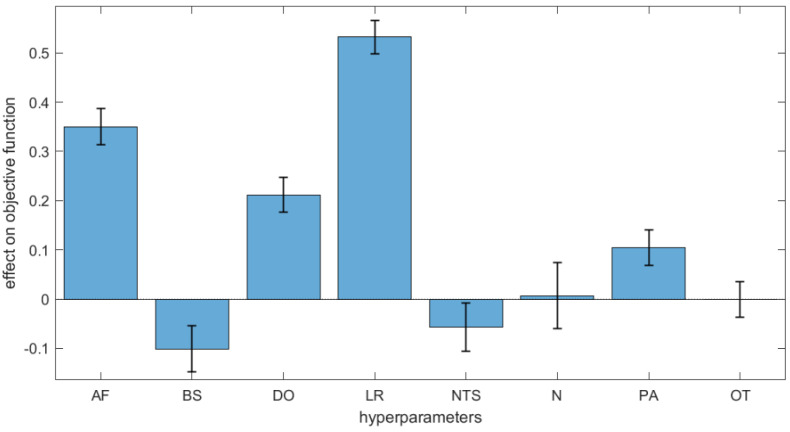
Bar plot of the effects of the ANNs hyperparameters on the objective function with their 95% confidence intervals. AF: activation function; BS: batch size; DO: dropout; LR: learning rate; NTS: number of task-specific neurons; N: number of neurons; PA: patience; OT: optimisation type.

The effect of each hyperparameter in terms of modelling capability was evaluated by means of a DoE approach [42]. The hyperparameters were used as the independent variables and the objective function *F* (Equation (5)) as the response through multivariate regression. The regression standardised coefficients measure the effects of the hyperparameters on the objective function and thus their contribution to the network output. As shown in Figure 2, Learning Rate (LR) and Activation Function (AF) are the most influential settings among the hyperparameters with positive effect. Dropout (DO) has a less relevant effect, while the other hyperparameters have no or very little effects. For these hyperparameters (Table 2) we chose the most convenient levels; for example, we set the number of neurons (N) to low levels to decrease the computational time, since this hyperparameter demonstrated to have very small effect on the modelling outcomes.

### 3.2. Model Performance and Similarity Matching

We evaluated the capability of our multi-task model to accurately predict the molecular fingerprint from the MS spectrum of a compound, both in terms of *NER*, which is related to the balanced accuracy of prediction of bits, and average Jaccard–Tanimoto similarity (*JT*) between the true and predicted fingerprints. Table 3 collects *NER* and *JT* values achieved on the training, validation and test sets for each of the two analysed datasets (12K and 40K), considering the different feature representations, that is, the raw 6596 *m*/*z* values and the SPCA scores.

The overall quality of the trained networks is quite similar and moderate differences can be observed when comparing *NER* and *JT* values. The effect of data curing (deepened in the 12K dataset) is by some means evident in the *JT* similarities obtained on the training set, which ranges from 0.61 and 0.58 on the 12K dataset to 0.50, 0.54 and 0.56 on the more extended 40K dataset. However, this trend is attenuated on the test set, where *JT* similarity reached maximum values equal to 0.47 and 0.45 for the 12K and 40K datasets, respectively. Therefore, quality input data support the learning of ANNs, but the improvement in terms of fingerprint similarity when predicting new chemicals was not significant to justify the use of a very small dataset for training. In fact, the training based on limited data can theoretically reduce the applicability domain and the generalisation ability of the models when using these methods to predict structures on very extensive databases.

The effect of data dimensionality reduction with the use of SPCA components as input features led to better results, even if also, in this case, the difference with respect to the predictive ability of the networks trained on the full spectra is very small. For example, networks trained on the 40K dataset with 500 input SPCA components provided *JT* similarity on the test set equal to 0.45, while for the full spectra *JT* was equal to 0.43. For the 40K dataset, the dimensionality reduction based on just 500 components (84.3% explained variance) did not affect the overall modelling quality and actually the solution based on 500 components provided the best matching between true and predicted fingerprints (i.e., *JT* of 0.45). Therefore, the data reduction based on SPCA can suggest new approaches for modelling MS data, especially when dealing with more extended datasets for network training.

**Figure 3:**
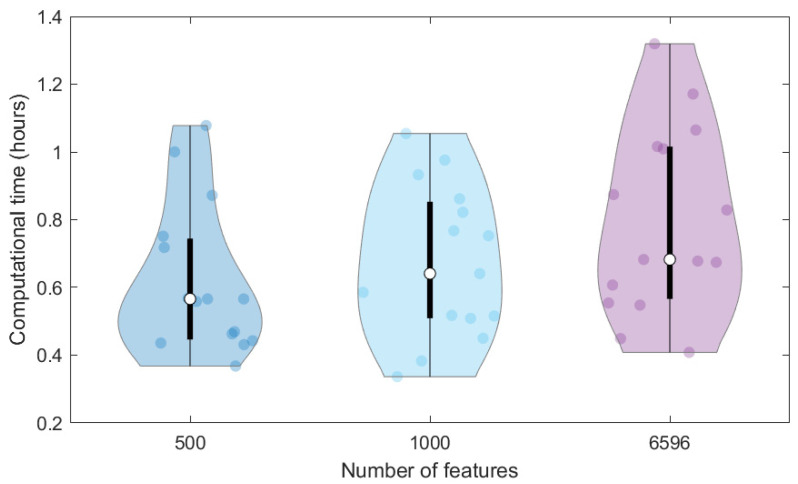
Violin plot of computational times required to train replicates of ANNs with 500 SPCA scores, 1000 SPCA scores and the 6596 raw MS features for the dataset 40K.

In addition, we compared the computational time required to train the same ANN on the 40K dataset represented through different features, that is, the original MS spectra (6596 signals) and the SPCA features (1000 and 500 scores). To avoid the effect of the initial random initialisation of weights on the computational time, we repeated calculations 15 times (replicates) and represented the computational times in the violin plot of Figure 3. The higher the data dimensionality, the higher the variation of computational times among the replicates and the higher the average time required for the network training, which varies from 0.62 to 0.67 and 0.79 h for 500, 1000 and 6596 dimensions, respectively. Therefore, the use of SPCA scores can also moderately reduce the time needed for network training, which can however have a more relevant effect when considering the large number of architectures to be tested in the tuning phase.

Looking at the overall results achieved on the training, validation and test sets of Table 3, the models demonstrated to be sufficiently accurate in terms of *NER*, being all values sufficiently higher than the benchmark *NER* of 0.5, which is associated with random classification [41]. However, the actual objective of these models is the similarity matching between true and predicted fingerprints; thus, we also compared our results versus random results in terms of *JT* similarity. For each compound in the test set of the 40K data, we generated 10 fingerprints by randomly selecting the bit values (0 or 1) with the constraint that the fractions of active bits (bits = 1) were maintained. Then, we calculated the *JT* similarity between the true fingerprint and the 10 randomly generated binary vectors for all the test compounds obtaining average *JT* similarity values from 0.03 to 0.24. These values are considerably lower than the average similarity obtained through the multi-task ANNs (0.45) and, thus, we can conclude that the proposed modelling approach learns useful structural information from the MS spectra.

Finally, we evaluated the network outputs also in terms of stability through replicates, as a measure of reliable predictions. As described in the method section, we replicated network calculations five times to avoid the effect of random initialisation of weights and predictions were then obtained by means of a majority voting approach. We estimated the variability among replicates by calculating, for each bit of the predicted fingerprints, the standard deviation and then we obtained the average value over the entire fingerprint vector and the entire set of fingerprints. Average standard deviations resulted in a range between 0.02 and 0.04, depending on the modelled dataset. However, these values correspond to very low fluctuations among replicates, indicating a very good agreement between the fingerprints predicted in the replicates.

### 3.3. Diagnostic for the Matching of Experimental and Predicted Fingerprints

The fingerprints predicted by the proposed multi-task ANNs were further analysed to understand the specific chemical features responsible for the matching between predicted and true fingerprints. To this end, we considered the predictions from the 5233 spectra included in the test set by means of the networks trained on the extended dataset (40K) over the 500 SPCA scores, that is, the model giving the best overall matching between fingerprints in terms of *JT* similarity (0.45, Table 3).

**Figure 4:**
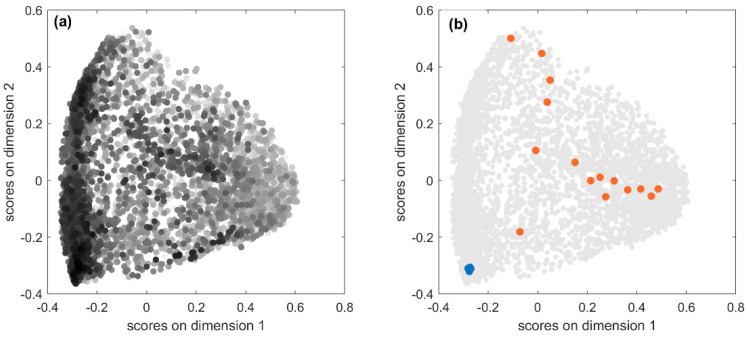
Score plot of the first and second MDS dimensions for the 40K test set predicted fingerprints; (**a**) fingerprints are coloured in a greyscale, the higher the similarity between predicted and true fingerprint, the darker the colour; (**b**) predicted fingerprints of exemplificative chemicals are coloured in blue (high accuracy between predicted and experimental fingerprints) and orange (low accuracy).

Diagnostics were carried out by calculating pairwise molecular similarities between predicted fingerprints using the Jaccard–Tanimoto similarity coefficient, which were then used to produce a two-dimensional representation by means of Multidimensional Scaling (MDS) of the predicted molecular space [53]. In Figure 4a, the score plot of the first two MDS dimensions is shown: each point represents one of the 5233 predicted fingerprints, near points indicate similar predicted fingerprints, while far apart points indicate very dissimilar fingerprints. Fingerprints are coloured in a greyscale, the higher the similarity to the true fingerprint, the darker the colour. Therefore, looking at the point distribution in the MDS plot, it can be seen that well-predicted structures were mainly clustered in the left-bottom corner. This may indicate the existence of some relationships between prediction accuracy and chemical features. To better understand this issue, we further explored results.

The test set comprises 5233 spectra associated with 711 unique chemicals, so one would expect less scattering when looking at Figure 4a. Actually, the fingerprints predicted for the same chemical from different spectra measured in different experimental conditions can differ in one or more bits. Hence, the relationship between true and predicted fingerprints is usually of type one-to-many and not all the predicted fingerprints have the same level of accuracy. Accuracy is expected to be higher in the chemical space regions populated by dark points, that is, predicted fingerprints associated with high *JT* similarity, and the opposite in the regions where the light points are more abundant. In order to verify this condition, we considered the predicted fingerprints for two compounds with opposite outcomes in terms of fingerprint accuracy. Figure 4b highlights in blue the 20 fingerprints predicted from the MS spectra of Kaempferol-3-O-robinoside-7-O-rhamnoside, which are located in the cluster with high accuracy and characterised by high *JT* similarities to the true fingerprint (average *JT* equal to 0.92). As a consequence, all these predicted fingerprints are also very similar to each other meaning that they encode very similar chemical structural features. On the opposite, the orange points represent 16 fingerprints predicted for 3-hydroxy-3-methylglutarate, which had extremely low *JT* similarities to the true fingerprint (average equal to 0.09). These fingerprints are scattered over different regions of the chemical space, thus denoting low similarity to each other, which means that they encode very different molecular structures and therefore are less effective for the identification of a compound from its MS spectra.

**Figure 5:**
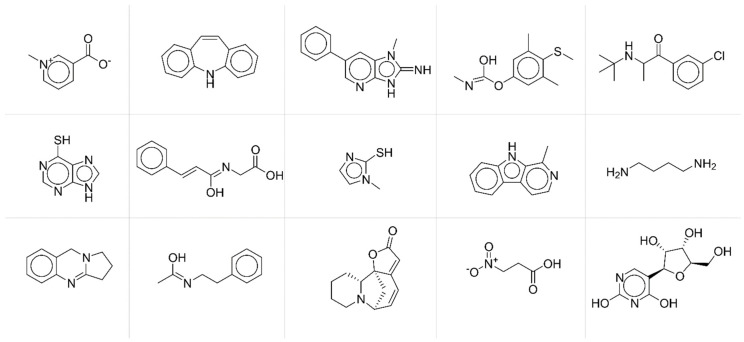
Exemplificative chemicals with low accuracy between predicted and true fingerprints.

**Figure 6:**
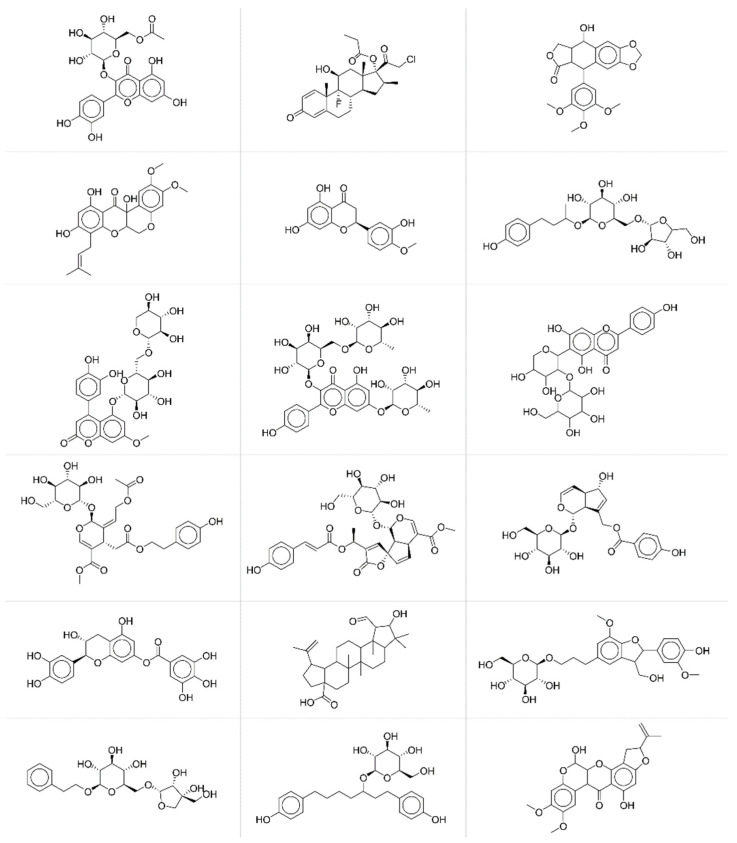
Exemplificative chemicals with high accuracy between predicted and experimental fingerprints.

To deeper investigate the potential relationships between modelling accuracy and chemical structural features of compounds, we collected compounds with similar behaviours in terms of fingerprint accuracy. More specifically, we extracted from the test set compounds with average *JT* similarity between predicted and true fingerprints lower than 0.25 (Figure 5) and chemicals with average *JT* similarity higher than 0.75 (Figure 6).

By a simple visual inspection and comparison of the two groups of molecular structures, it is apparent that chemicals associated with higher prediction accuracy are characterised by more complex molecular structures and thus by more informative MS spectra. Thus, the accuracy in the fingerprint matching could be associated with the complexity of the molecular structure, the higher the complexity, the better the prediction. Complexity of molecular structures is somehow related to the number of active bits (=1) in the fingerprints. The higher the structural complexity, the more the structural features to be encoded and thus the higher the number of active bits in the fingerprint, the better the prediction. ANNs learn better the information when input (MS spectra) and output (fingerprints) vectors are less sparse.

**Figure 7:**
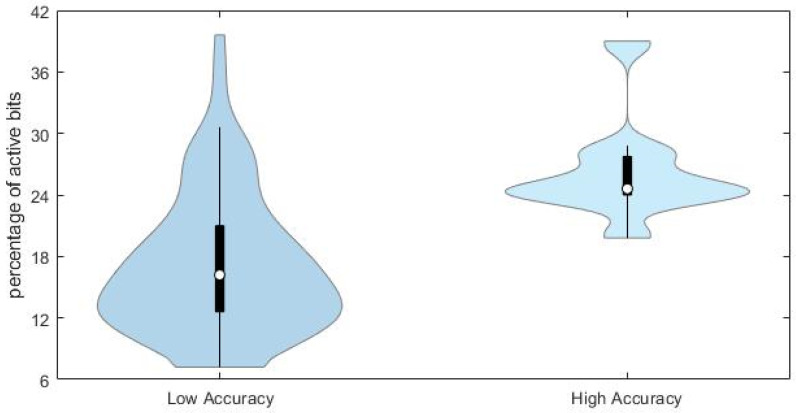
Violin plot showing the distribution of the percentage of active bits in the fingerprints of chemicals with low and high accuracy of predicted fingerprints.

This conclusion is supported by the comparison of the distributions of the percentage of active bits in the true fingerprints for the compounds with high (*JT* similarity > 0.75) and low (*JT* < 0.25) accuracy in the predicted fingerprints, as shown in Figure 7.

Chemicals with lower prediction accuracy have significantly lower percentage of active bits in the fingerprints (median value equal to 17%, against 24% for those chemicals with higher prediction accuracy). Results may suggest that the prediction accuracy and the subsequent similarity matching can be enhanced when taking into account specific portions of the applicability domain of the model, especially those related to higher complexity of molecular structures.

## 4. Conclusions

In this study, we developed a novel multi-task model to predict molecular fingerprints from LC-MS/MS data with the aim to provide fast automated tools to support the identification of metabolite structures. MS spectra from the MassBank of North America repository were collected, cured and used as inputs for the training of multi-task ANNs. Sparse Principal Component Analysis was applied to reduce MS data dimensionality and evaluate the effect of data sparseness on the modelling accuracy. The molecular structures of the compounds in analysis have been encoded by the MACCS keys fingerprints, which were used as the output for the network’s training. Data were divided in training, validation and test sets, avoiding the selection of the spectra associated with the same compound in different sets to stress the validation conditions.

ANN architectures were tuned through the TPE approach by leveraging similarity matching between true and predicted fingerprints, as well as the reduction of potential overfitting. The overall quality of the trained networks was considered acceptable, with results significantly higher than those of randomly generated fingerprints. In particular, balanced accuracy (Non-Error Rate, *NER*) from 0.69 to 0.70 and average Jaccard–Tanimoto similarity between true and predicted fingerprints from 0.43 to 0.47 were achieved on the test set.

Deepened data curing produced better results when looking at training and validation sets, but this trend was diminished on the test set. Therefore, quality input data supported the network’s learning, but the increase in terms of fingerprint similarity when predicting new molecules did not justify the use of a smaller dataset for training, which can reduce the model applicability domain.

The effect of dimension reduction with the use of SPCA led to better results, but with a very small difference with respect to what was achieved by means of the full MS spectra. However, data dimensionality reduction of the input MS data can contribute to determining the decrease in computational time.

Finally, when exploring relationships between the model accuracy and structural features through the analysis of the fingerprint chemical space, we found that accuracy of predicted fingerprints can be associated with the complexity of molecular structures, the higher the complexity, the better the prediction. This can support the conclusion that ANNs learn better the information when input spectra and fingerprints are less sparse.

## Figures and Tables

**Table 1:** Number of MS spectra and compounds included in the training, validation and test sets for the 12K and 40K datasets.

	40K Dataset		12K Dataset	
	MS Spectra	Compounds	MS Spectra	Compounds
Training	29,279	4040	9037	2804
Validation	6059	806	1828	577
Test	5233	711	1685	501
Total	40,571	5557	12,550	3882

**Table 2:** Tested levels and optimal values of ANNs hyperparameters for the architectures trained with the 12K and 40K datasets.

	Minimum and Maximum Level	Optimal Hyperparameters 40K Dataset	Optimal Hyperparameters 12K Dataset
Number of neurons (N)	50–100	100	100
Neurons task-specific (NTS)	250–500	250	500
Learning rate (LR)	0.0001–0.01	0.006	0.0025
Activation function (AF)	Sigmoid, ReLU	Sigmoid	Sigmoid
Dropout (DO)	0–0.5	0.30	0.33
Batch size (BS)	2000–4000	2000	600
Patience (PA)	50–150	95	114
Optimisation type (OT)	Adam, SGD, RMSprop	RMSprop	RMSprop

**Table 3:** Non-Error Rate (*NER*) and average Jaccard Tanimoto similarity (*JT*) achieved on the training, validation and test sets with the ANNs trained on the 12K and 40K datasets with different number of features.

			Train		Validation		Test	
Dataset	Spectra	Features	*NER*	*JT*	*NER*	*JT*	*NER*	*JT*
40K	40,571	1000	0.82	0.56	0.69	0.48	0.69	0.44
40K	40,571	500	0.81	0.54	0.69	0.48	0.69	0.45
40K	40,571	6596	0.79	0.50	0.69	0.45	0.70	0.43
12K	12,550	500	0.84	0.61	0.70	0.49	0.69	0.47
12K	12,550	6596	0.82	0.58	0.70	0.47	0.70	0.46

## Data Availability

The dataset is available for download at the Milano Chemometrics and QSAR Research group https://michem.unimib.it/download/data/lc-ms-ms-to-fingerprints-dataset (accessed 4 September 2022).
